# A Helpful Book: Its Story and Results

**Published:** 1916-08-05

**Authors:** 


### THE INSTITUTIONAL LIBRARY.

### A Helpful Book: Its Story and Results.

**Burdett's Hospitals and Charities, 1916.** By Sir Henry Burdett, K.C.B., K.C.V.O. (London: The Scientific Press. Price 10s. 6d. net.)

The title-page of this work describes it as an exhaustive record of hospital work for the year, and the most useful and reliable guide to British, American, and Colonial hospitals and asylums, medical schools and colleges, nursing and convalescent institutions, consumption sanatoria, religious and benevolent institutions, and dispensaries." Anyone who, like the writer, has constantly to refer to the book will readily admit that it is no empty claim. The directory portion alone contains particulars of about 6,000 institutions of all kinds, and of this portion it is enough to say that its compilation reflects the highest credit upon those responsible. It is, however, in the introductory chapters that the special and peculiar value of the Annual is to be found. These chapters would by themselves form a valuable reference book. They have not only an immediate but a permanent value, and the twenty-seven volumes of this book may fairly claim to constitute in themselves a reference library and history of the progressive development of voluntary charities. A new and useful feature of the present volume is the inclusion under the Medical Schools and University section of a list of post-graduate colleges, with particulars of fees, lectures, and demonstrations.

Of the special articles in the present volume two deserve mention as being timely and instructive. One is entitled "The Great War," and deals, *inter alia*, with the effect of the war upon the voluntary hospitals and their administration. The other, entitled "The Volume of Charity," is a painstaking piece of statistical work showing in detail the income and expenditure for 1913 and 1914 of nearly 2,000 charitable institutions in the British Isles. It is not a little surprising to find that, in spite of the demands of all kinds which the war has made upon the charitable public, the total income for 1,988 societies in 1914 was £13,630,000, against £13,947,500 for 2,122 institutions in 1913, and the expenditure was £12,474,500, against £12,642,800 for 1913. That the expenditure for 1915 will be maintained at anything like this figure is scarcely to be looked for, seeing that the 1914 expenses were largely the result of contracts entered into before the war began. The following figures as to the maximum and minimum prices paid by London hospitals for various commodities during 1914 are interesting, but, unfortunately, they do not furnish a standard for the guidance of purchasers at the present moment: Top sides of beef, per lb., highest, 1s.; lowest, 4¾d. Legs of mutton, per lb., highest, 11d.; lowest, 5d. Fish, per lb., highest, 10d.; lowest, 2½d. Bacon, per lb., highest, 1s. 2d.; lowest, 5½d. Milk, per gallon, highest, 1d. 4d.; lowest, 7$\frac{13}{16}$d. Bread, per 4 lb., highest, 8d.; lowest, 4d. Potatoes, per cwt., highest, 8s.; lowest, 3s. 2d.

The tables as to the hospital income and expenditure are not only models of statistical compilation, but they have undoubtedly had an educational influence upon those from whom the figures are obtained. But for Sir Henry Burdett's insistence, year after year, upon the importance of a Uniform System of Accounts, it is doubtful whether the adoption of the Uniform System would have become so general in the provinces. In the volume for 1895 there were 160 hospitals included in these tables, of which only sixty-three had adopted the Uniform System. In the tables in the present volume the number of hospitals dealt with is 165, of which only seventeen have not yet fallen into line. As showing the thoroughness with which these tables are compiled and the importance which the Editor attaches to their accuracy, it may be mentioned that the writer has for many years made very frequent and critical use of the tables, and on one occasion only (in 1906) does he remember to have discovered a mistake. On this mistake being pointed out to the Editor, a reprint of the page containing the mistake was immediately issued to all the subscribers.

On looking through back volumes of the Annual one is reminded that in order to arrive at the average cost of each in-patient the Editor used to deduct from the total expenditure a fixed amount per out-patient, varying, according to the class of hospital, from 1s. 6d. to 5s. This method was admittedly open to criticism, and has now been abandoned, Sir Henry Burdett having succeeded, after many years of effort, in persuading the authorities of the leading hospitals to keep separate accounts for the in- and out-patient departments. In consequence, through the co-operation of the authorities of 131 out of the 165 hospitals included in the tables, he is able to publish figures showing the total expenditure after deducting (1) rent, rates, and taxes, and (2) the cost of the out-patient department. There can be no question that the separation of the accounts of the two departments must make for greater accuracy in Sir Henry Burdett's calculations, provided the items are apportioned upon an equitable basis; but the table on page 99, giving the cost per out-patient at these 131 hospitals, justifies the suspicion that uniformity in this matter has by no means yet been reached. For example, the cost per out-patient at King's College Hospital is given as 3s. 9½d., against only 2s. 4d. at the Middlesex Hospital. Of the provincial hospitals with medical schools each out-patient at the Birmingham General Hospital cost 2s. 6d., as compared with only 1s. 2½d. at the Royal Victoria Infirmary at Newcastle. At the children's hospitals the cost ranged from 3s. 5.7d. at the Hospital for Sick Children, Great Ormond Street, to 1s. 5½d. at the East London. Amongst hospitals for consumption the variations are even more remarkable, ranging from 15s. 2d. per out-patient at the Brompton Hospital to 5s. 3d. at the City of London. There would appear to be no special reason why there should be such variations as these amongst hospitals of a similar character, and an inquiry by Sir Henry Burdett would probably provide useful matter for a chapter in a future volume. The only disappointing chapter in the book is that relating to Indian, Colonial, and American hospitals, where the Editor makes the following quaint remark at the head of one of the tables: "The remarkable feature of this and the expenditure tables is that so many blank spaces should be shown against United States hospitals. The returns from these hospitals cannot all have been sent to the bottom of the sea by a torpedo, can they?"

The book is well printed and well indexed. The test of constant use has placed its excellence and accuracy beyond question, and we can only add that, while the book ought to form part of the reference library of every business or public man, to those who are concerned in any way with the administration of charitable institutions it has become almost indispensable.

